# Is It Possible to Reverse the Storage-Induced Lesion of Red Blood Cells?

**DOI:** 10.3389/fphys.2018.00914

**Published:** 2018-07-24

**Authors:** Gregory Barshtein, Dan Arbell, Leonid Livshits, Alexander Gural

**Affiliations:** ^1^Faculty of Medicine, Biochemistry Department, Hebrew University of Jerusalem, Jerusalem, Israel; ^2^Pediatric Surgery, Hadassah-Hebrew University Medical Center, Jerusalem, Israel; ^3^Institute of Veterinary Physiology, University of Zurich, Zürich, Switzerland; ^4^Blood Bank, Hadassah-Hebrew University Medical Center, Jerusalem, Israel

**Keywords:** red blood cells, rejuvenation, erythrocyte membrane, aging, blood banks, transfusion, deformability, fragility

## Abstract

Cold-storage of packed red blood cells (PRBCs) in the blood bank is reportedly associated with alteration in a wide range of RBC features, which change cell storage each on its own timescale. Thus, some of the changes take place at an early stage of storage (during the first 7 days), while others occur later. We still do not have a clear understanding what happens to the damaged PRBC following their transfusion. We know that some portion (from a few to 10%) of transfused cells with a high degree of damage are removed from the bloodstream immediately or in the first hour(s) after the transfusion. The remaining cells partially restore their functionality and remain in the recipient’s blood for a longer time. Thus, the ability of transfused cells to recover is a significant factor in PRBC transfusion effectiveness. In the present review, we discuss publications that examined RBC lesions induced by the cold storage, aiming to offer a better understanding of the time frame in which these lesions occur, with particular emphasis on the question of their reversibility. We argue that transfused RBCs are capable (in a matter of a few hours) of restoring their pre-storage levels of ATP and 2,3-DPG, with subsequent restoration of cell functionality, especially of those properties having a more pronounced ATP-dependence. The extent of reversal is inversely proportional to the extent of damage, and some of the changes cannot be reversed.

## Introduction

Red blood cell (RBC) transfusion is a life-saving procedure whose primary objective is to sustain tissue and organ oxygenation in patients with massive bleeding or acute anemia. Packed red blood cell (PRBC) donations for transfusion are routinely stored for up to 35 or 42 days, depending on the preservation solution. This time limit has been determined mainly by the recovery and the lifespan of the RBCs in the circulation of recipients.

During cold-storage, PRBCs undergo slow detrimental changes that are collectively termed storage lesion. Oxidative-stress and defective adenosine triphosphate (ATP) metabolism are the main driving forces in the development of PRBC lesion. Storage-related processes lead to significant metabolic and structural changes of erythrocytes that include global biochemical and biophysical alteration, remodeling of the cell membrane and cytoplasm composition (Hess, 2010; Obrador et al., 2015).

The most studied changes include: (ATP) and 2,3-bisphosphoglycerate (2.3-DPG) depletion (Hess, 2010), loss of cellular antioxidant capability (Racek et al., 1997; Dumaswala et al., 2000), changes in K^+^ and Na^+^ concentration (Olivieri et al., 1993; Cicha et al., 2000), loss of membrane’ and skeleton proteins (Ciana et al., 2017; Orbach et al., 2017), loss of membrane lipids and changes in their in/out distribution, vesicle generation (Bosman et al., 2008, 2010), oxidation and remodeling of skeleton proteins (Wolfe et al., 1986), clustering of band 3 proteins (Pantaleo et al., 2008; Arashiki et al., 2013), alteration of nitric oxide signaling (Almac et al., 2014; Liu et al., 2014), decrease in antioxidant activity (Dumaswala et al., 1999; Dzik, 2008; Whillier et al., 2011) etc.

A number of these changes are interrelated and initiate a cascade of biochemical and structural changes which in turn lead to impairment in RBC functionality, specifically – alteration in the biophysical/mechanical properties of cells.

Each of the reported storage-induced alterations in PRBC properties occurs on its specific time-scale (d’Almeida et al., 2000; Bennett-Guerrero et al., 2007; D’Amici et al., 2007; Relevy et al., 2008; D’Alessandro et al., 2012; Gevi et al., 2012; Santacruz-Gomez et al., 2014; Kozlova et al., 2015; Bardyn et al., 2017). Some of these changes take place at an early stage of storage (during the first 7 days), while others occur later. Prudent et al. (2015) have extensively discussed this issue, distinguishing between the separate phases of PRBCs lesion development and providing a detailed scheme of the various changes on a time-scale. Different stages of cell aging are also reported by Nishino et al. (2009, 2013) and Gevi et al. (2012).

When a patient is being transfused with a PRBCs unit, cells with a specific set of features are administered into his bloodstream. Senescent transfused cells, with a high degree of damage, are removed from the bloodstream shortly following the transfusion (Hunsicker et al., 2018). Furthermore, we have recently demonstrated a dependence of transfusion-induced hemoglobin increment (determined upon completion of the transfusion) on the percentage of low-deformable cells in the population of the transfused RBCs (TRBCs) unit (Barshtein et al., 2017). We suggested that during the transfusion the relatively rigid cells in the TRBCs population (composing dozens of percentages) are immediately removed itself. This suggestion is in accord with previous reports indicating that the clearance of TRBCs starts directly with the administration of cells into the bloodstream (Luten et al., 2008; Bosman, 2013).

Rigid and fragile RBC are subject to facilitated removal from circulation within the first hours after transfusion, due to shear forces operating in the bloodstream (Nagababu et al., 2016). Thus, Luten et al. (2008) concluded that significant portion of the TRBCs that do not survive the first 24 h are removed from the circulation within the first few hours after transfusion.

Following removal of the extremely damaged cells, most of the TRBCs [in the range of 75% (Luten et al., 2008)] remain in the bloodstream of the recipient in an environment substantially different from the one in which they were stored. As a result, the remaining TRBCs can partially restore their functionality and to remain in the recipient’s blood for a more extended period. The average lifespan of transfused RBCs is about 50–60 days, but it can be significantly shorter in the presence of factors reducing their survival (Liumbruno et al., 2009).

Accordingly, an assessment of reversibility of the cold-storage lesion is a critical issue in the field of RBCs storage and transfusion as the ability of transfused RBCs to restore their properties following transfusion. This ability largely determines TRBCs behavior in the bloodstream of the recipient.

In the present review, we discuss publications that examined RBC lesions induced by the cold-storage, aiming to offer a better understanding of the time frame in which these lesions occur, with particular emphasis on the question of their reversibility. Also, we propose an approach for assessing the potential for reversibility of the damage to specific properties of PRBCs.

## Post-Transfusion Behavior of RBCs

Of all the properties of TRBCs, the ability to restore of ATP and 2,3-DPG levels is most extensively discussed in the literature. Since some physiological factors can modify the time course of the post-transfusion restoration, the reported time needed for full recovery varies widely from several hours to several days (Valtis, 1954; Beutler and Wood, 1969; Valeri and Hirsch, 1969; Enoki et al., 1986; Heaton et al., 1989). The ATP level in TRBCs rapidly increases in the recipient’s bloodstream (Valeri and Hirsch, 1969; Heaton et al., 1989). Valeri and Hirsch (1969) suggested that this effect is associated with a rapid decrease in the donor cell Na^+^ content during the 24 h post-transfusion period. In parallel, TRBC cellular K^+^ content is gradually increased, with the rate of this increase being related to the increasing level of intracellular 2,3-DPG (Valeri and Hirsch, 1969).

Heaton et al. (1989) measured the levels of 2,3-DPG and ATP in transfused cells for 1 week following the transfusion. The authors demonstrated that an average of 95% of the recipients’ pre-transfusion 2,3-DPG level was reached by 72 h, while ATP level in started increasing 1 h after transfusion (Heaton et al., 1989). D’Alessandro et al. (2010) have reported that the repairing of Na^+^/K^+^ pump activity occurred during 1 to 4 days.

In contrast to the data presented so far, some publications (Frank et al., 2013; Chadebech et al., 2016, 2017) have reported a deterioration in the TRBCs properties following their interaction with human plasma. Thus, Chadebech et al. (2016, 2017) demonstrated that an *ex vivo* incubation of PRBCs with the plasma of healthy subjects (Chadebech et al., 2016, 2017), patients with sepsis (Chadebech et al., 2017) and sickle anemia patients (Chadebech et al., 2016) induced alterations in the phosphatidylserine (PS) externalization and a decrease in the average cell size.

Frank et al. (2013) measured deformability of RBCs from post-surgery patients prior to and following a moderate (≥five units) or a minimal (≤four units) PRBC transfusion. They demonstrated that deformability of the patients’ RBCs has decreased following transfusion as compared to pre-transfusion cells, and this abnormality was not reversed and even got worse during the subsequent 3 days.

## What Lesion Is Reversible?

Which are the properties of the stored PRBCs that we can expect to be corrected, and which ones are not? This question is extensively discussed in the literature (Beutler et al., 1982; Luten et al., 2008; D’Alessandro et al., 2010; Prudent et al., 2015). For example, D’Alessandro et al. suggest that the reversibility of these changes is inversely proportional to the duration of storage (D’Alessandro et al., 2010). Prudent et al. takes a similar position (Prudent et al., 2015), with the authors concluding that an “early” lesion (i.e., one that occurs following first 2 weeks of storage) is reversible, while a “late” lesion (4 weeks and more) is not. This suggestion is supported by *in vivo* studies of TRBCs performed by Beutler et al. (1982) and Luten et al. (2008), who conclude that RBC storage is associated with irreversible damage that increases with storage duration.

## Do Any Irreversible Changes Occur in the Early Stages of Storage?

During the first week of storage, RBC lesion is characterized by the formation of micro-defects in the RBC membrane and a decrease in the membrane roughness, as assessed by atomic force microscopy (Girasole et al., 2012; Pompeo et al., 2012; Kozlova et al., 2015; Acosta-Elias et al., 2017). For example, Kozlova et al. (2015) demonstrated that by days 9 to 12 topological defects in the form of “domains” appear on the membrane surface. These defects initially appear as grain-like structures (“grains”) of up to 200 nm (on days 16–23), and later merge to form large defects, 400–1000 nm. This observation conforms with that of Girasole et al. (2012) and Pompeo et al. (2012), who used atomic force microscopy to examine storage-induced structural and metabolic changes in PRBCs. The authors demonstrated that the RBC roughness might be restored to the initial value only in samples stored for up to 4–5 days, whereas after the eighth day of storage the rejuvenation procedure appears to be inefficient. Girasole et al. (2010, 2012) concluded that some changes, localized on the membrane surface, appear when the ATP synthesis capacity declines to apparently sub-viable levels and the membrane-skeleton is permanently damaged (Pompeo et al., 2012).

Moreover, irreversible reduction in surface charge (Godin and Caprani, 1997; Silva et al., 2012; Erman et al., 2016) has been observed under different conditions of RBC *in vitro* aging. Specifically, Silva et al. (2012) examined the effect of cold-storage of human RBCs (collected into CPD-SAGM, leukodepleted and unleukodepleted) on the ξ-potential of the cell membrane, and demonstrated that the ξ-potential of cells decreases during the first week of storage. The same authors (Silva et al., 2012) have also demonstrated that ξ-potential decay (30%) of RBC samples (leukodepleted and not) was more pronounced during the first week of storage, suggesting that this decay is caused by oxidative damage generated by the production of reactive oxygen species.

The authors suggest that the decrease in RBC deformability observed during cell aging (*in vivo*) (Huang et al., 2014) and cold storage (Silva et al., 2012) could arise from a reduction of their ξ-potential.

Moreover, a release of microvesicles from RBC membrane starts at the second week of cold-storage. These vesicles are characterized by a significant fraction of lipid raft proteins [such as stomatin and flotillins (Salzer and Prohaska, 2001)] as well as oxidized or reactive signaling components commonly associated with senescent RBCs. Kriebardis et al. (2008) demonstrated that the vesicular protein content progressively increased over time, with a twofold increase in PRBCs stored for 17 days as compared to those stored for 7 days.

The formation of microvesicles leads to a decrease in the content of membrane proteins (Salzer and Prohaska, 2001; Wilkinson et al., 2008; D’Alessandro et al., 2012) and a change in the composition of the cytoskeleton. Thus, Ciana et al. (2017) suggested that part of the membrane skeleton is lost together with components of the lipid bilayer in a balanced way.

Song et al. (2017) demonstrated that during cold-storage the protein components of RBC skeleton are partially decomposed. The thickness of the cell membrane is reduced during storage and, specifically, shows a sharp decrease from the 6^th^ to the 14^th^ day of cold-storage.

Mechanism of vesiculation is discussed in detail in numerous publications (D’Alessandro et al., 2015; Antonelou and Seghatchian, 2016; Ciana et al., 2017). Nonetheless, the selective sorting of specific membrane components into microvesicles remains to be understood. In any case, regardless of the mechanism, the formation of vesicles leads to irreversible changes in the composition and structure of the membrane. Specifically, it induces a decrease in the area-volume ratio, which affects the cells’ shape, their mechanical properties (Svetina, 2012) and survival in the bloodstream (Deplaine et al., 2011).

Thus, some basic cells properties are irreversibly altered during the first to third week of storage. These changes necessarily produce alterations in the functionality of the transfused erythrocytes, some of which cannot be fully reversed following transfusion (Barshtein et al., 2014).

## Post-Storage Rejuvenation Treatment *In Vitro*

One of the possible procedures proposed for the reversal of storage-induced damage is post-storage rejuvenation treatment, which consists of supplementing PRBC units with rejuvenation solution before their transfusion (Kiartivich et al., 1986; Lockwood et al., 2003; Verhoeven et al., 2006; Raat et al., 2009). A commercially available rejuvenation solution is produced under trademark Rejuvesol and consists of sodium pyruvate, inosine, adenine, and sodium phosphate. The FDA approves this solution and, although rarely applied in clinical blood banking, is widely used in research for restoring cellular ATP and 2,3-DPG levels (d’Almeida et al., 2000; Yoshida et al., 2008; Meyer et al., 2011). Of course, the research results should be interpreted with caution as an *in vitro* method with significant limitations for clinical interpretation.

The rejuvenation procedure can be carried out at 37°C (“warm”) and 4°C (“cold”), with the “warm” type being more extensively studied than the cold one. Still, a comparison of the results reported by different authors (d’Almeida et al., 2000; Koshkaryev et al., 2009; Tchir et al., 2013; Barshtein et al., 2014; Kurach et al., 2014) leads to the conclusion that rejuvenation is more effective when performed at 37°C. In the following section, we will only consider results obtained with a warm treatment, which is consistent with the objective of the present review.

Indeed, during previous attempts to attenuate the impairment of PRBCs functionality caused by cold-storage, it was shown that rejuvenation treatment could restore intra-cellular ATP and 2,3-DPG levels and thus reverse a storage-induced alteration to various properties of cells (Dumaswala et al., 1992; Koshkaryev et al., 2009; Barshtein et al., 2014). Specifically, rejuvenation leads to repairing of PRBCs morphology (Usry et al., 1975) and mechanical properties: deformability (Barras et al., 1994; d’Almeida et al., 2000; Barshtein et al., 2014) and fragility (DeVenuto et al., 1974; Gelderman and Vostal, 2011; Barshtein et al., 2014), although, as could be expected, this positive effect decreases with an increasing storage duration (d’Almeida et al., 2000; Meyer et al., 2011).

Importantly, the effect of rejuvenation on stored PRBCs is not limited to the restoration of intracellular levels of ATP and 2,3-DPG: it was shown that treatment of stored cells by Rejuvesol had induced significant metabolic reprogramming, including reactivation of energy-generating and antioxidant pathways, and membrane lipid recycling (D’Alessandro et al., 2017).

## Reversibility of PRBC Lesion *In Vitro*

In this section, we will focus on the reversibility of specific functional properties of cells that determine their behavior after transfusion to the patient, especially – PS externalization and mechanical properties of their membrane.

The regulated plasma/membrane PS asymmetry is critical to many biological processes (Bratosin et al., 1998; Kuypers and de Jong, 2004; Lutz and Bogdanova, 2013; Dinkla et al., 2014). Thus, it has been shown that PRBCs storage initiates Ca^2+^ influx into cells with subsequent PS externalization and amplifies the susceptibility of TRBCs to eryptosis, the “suicidal” death (Lang et al., 2016). Furthermore, erythrocyte surface PS has been reported to activate coagulation and thus induce thrombosis (Chung et al., 2007; Shin et al., 2007; Gao et al., 2012).

PS externalization can be mediated by the decrease in the ATP level (Bitbol et al., 1987) and/or by activation of scramblase (Basse et al., 1996; Woon et al., 1999). Therefore, the storage-induced increase in intracellular [Ca^2+^] (Koshkaryev et al., 2009; Lang et al., 2016) may be sufficient to induce the observed elevation of PS-positive PRBCs via the activation of scramblase (Basse et al., 1996; Woon et al., 1999). Alternatively, Verhoeven et al. (2006) have reported that cold-storage did not activate scramblase (by Ca^2+^ influx), but rather inhibited flippase activity as a result of ATP depletion, which was subsequently reversed by the treatment with Rejuvesol (d’Almeida et al., 2000; Yoshida et al., 2008; Meyer et al., 2011). However, whatever the mechanism of action, since intracellular Ca^2+^ concentration is ATP dependent (Maher and Kuchel, 2003), the renewal of ATP content in PRBCs as a result of rejuvenation should lead to the restoring of phospholipids’ asymmetry (Maher and Kuchel, 2003).

In agreement with this assumption, Koshkaryev et al. (2009) have recently shown that the storage-induced alterations in PRBCs (elevation of [Ca^2+^], and PS externalization) are almost completely rectified by post-storage rejuvenation treatment, which caused a decrease in RBC adhesion to endothelial cells.

In discrepancy to this observation, in our publication (Barshtein et al., 2014) we demonstrated that treatment with the Rejuvesol causes only partial restoration of the cells’ mechanical properties (deformability and fragility).

To explain the difference in rejuvenation efficiency reported by different groups, we assumed that it is related to the degree of dependency of the specific property on the intracellular ATP concentration.

Indeed, it is well-known that various RBC functions and properties are mainly, if not solely, determined by the cell ATP level. Thus, it is plausible to expect the rejuvenation to be more effective in restoring cell properties that are strongly ATP-dependent.

To assess rejuvenation effectiveness (i.e., the degree of reversibility) we have formulated (Barshtein et al., 2014) rejuvenation effectiveness index (REI), describing the percentage of storage-induced damage to a specific RBC property that is restored by treatment.

In our publication (Barshtein et al., 2014) we summarized previously obtained results regarding reversibility of PRBC lesion and had shown that treatment of PRBCs with Rejuvesol had restored all tested properties. However, the extent of improvement varied considerably between the different measures, leading us to suggest that the REI is higher for features that are more ATP-dependent. Thus, for intracellular Ca^2+^ content and the level of PS externalization, REI is nearly 70%, with full recovery of RBC adhesion. In contrast, the rejuvenation efficacy in repairing the PRBC mechanical properties (deformability and fragility), was only 50%.

Of particular interest are the findings previously presented by us (Barshtein et al., 2014), showing that the efficacy of rejuvenation in the reversal of storage-induced impairment in the RBC mechanical properties depends on the extent of damage – thus, the higher the damage, the lesser the rejuvenation efficacy (Barshtein et al., 2014). Changes in RBC mechanical properties are often associated with irreversible structural alterations, such as changes in RBC surface-to-volume ratio due to vesiculation (Safeukui et al., 2012), or reduction in surface charge due to sialic acid degradation (Godin and Caprani, 1997; Huang et al., 2014), which are not expected to be affected by restoring the ATP content of RBC. These changes in the RBC membrane structure have been reported to increase with storage duration (Silva et al., 2012), thereby explaining why the rejuvenation efficacy is inversely related to the extent of storage-induced damage to PRBCs mechanical properties.

Thus, the ability of PRBCs to reverse (wholly or partially) their deformability and intracellular [2,3-DPG] content can explain the results obtained by Raat et al. (2009). The authors tested human PRBCs in an isovolemic transfusion model in rats after hemodilution. The cells were derived from units of PRBCs and transfused to animals with or without rejuvenation treatment before transfusion. It was demonstrated (Raat et al., 2009) that the rejuvenation of cells can completely reverse the reduced oxygenation capacity of long-stored human PRBCs in hemodiluted rats before transfusion.

## Limitations

In presented mini-review, we discussed the reversibility of PRBC lesion induced by cold-storage. For this purpose, we reviewed different studies examining PRBC recovery under *in vivo, ex vivo*, and *in vitr*o conditions. However, it is necessary to take into account the difference between the potential recoverability of a cell’s specific feature(s), and the possibility of its restoration after a PRBC transfusion to the patient.

## Future Direction

As demonstrated above, the reversibility of the RBC lesion *in vitro* (by incubation with Rejuvesol) has been extensively discussed in the cited literature. Unfortunately, this issue has been poorly studied under *in vivo* conditions, despite its theoretical and practical significance in assessing the role that storage lesion plays in transfusion outcome. Currently, PRBC units are supplied for transfusion on the first-in-first-out (FIFO) basis, assuming that their functionality is equal for all units with equal pre-transfusion storage duration. Thus, a number of large-scale clinical trials, both published (Hod et al., 2011; Steiner et al., 2015; Rapido et al., 2017; Stowell et al., 2017) and ongoing (Kekre et al., 2013), have focused their attention on the conditions of storage (specifically – on storage duration), and not on the properties of transfused RBCs and the reversibility of the storage lesion in the bloodstream. We believe that studying the PRBC lesion reversibility after transfusion should be the future direction of research on this subject, being of fundamental importance for assessing the survival of TRBCs in the bloodstream, and potentially leading to the improvement in the transfusion outcome.

## Conclusion

It is well-documented that the cold-storage of PRBC units is associated with a change in a wide range of RBCs properties, each of them taking place on its own specific timescale. Thus, even at an early stage of storage (the first or second week), irreversible changes in the properties of the cells can already occur.

Despite the fact that the behavior of TRBCs in the recipient’s bloodstream is still insufficiently studied, it can be argued at this stage that transfused cells are capable of restoring the pre-storage levels of ATP and 2,3-DPG, with subsequent restoration of cell functionality. This assumption is confirmed by the results of *in vitro* experiments demonstrating significant restoration of various PRBC features following incubation with a rejuvenation solution, especially those with a more pronounced ATP-dependence. The extent of reversal is inversely proportional to the extent of damage, and some of the changes cannot be fully reversed.

We hope that this review may stimulate further research into the field of cold storage and rejuvenation discussed here, and thus aid in the formulation of a new paradigm for PRBCs units inventory management.

## Author Contributions

GB, DA, LL, and AG have been involved in the analysis and discussion of studies relating to the subject and in the writing of the review.

## Conflict of Interest Statement

The authors declare that the research was conducted in the absence of any commercial or financial relationships that could be construed as a potential conflict of interest. The handling Editor declared a shared affiliation, though no other collaboration, with one of the authors LL at the time of the review.

## References

[B1] Acosta-Elias M. A. Burgara-Estrella A. J. Sarabia-Sainz J. A. Silva-Campa E. Angulo-Molina A. Santacruz-Gomez K. J. (2017). Nano alterations of membrane structure on both gamma-irradiated and stored human erythrocytes. *Int. J. Radiat. Biol.* 93 1306–1311. 10.1080/09553002.2017.1393581 29034757

[B2] Almac E. Bezemer R. Hilarius-Stokman P. M. Goedhart P. de Korte D. Verhoeven A. J. (2014). Red blood cell storage increases hypoxia-induced nitric oxide bioavailability and methemoglobin formation in vitro and in vivo. *Transfusion* 54 3178–3185. 10.1111/trf.12738 24942042

[B3] Antonelou M. H. Seghatchian J. (2016). Update on extracellular vesicles inside red blood cell storage units: adjust the sails closer to the new wind. *Transfus. Apher. Sci.* 55 92–104. 10.1016/j.transci.2016.07.016 27452642

[B4] Arashiki N. Kimata N. Manno S. Mohandas N. Takakuwa Y. (2013). Membrane peroxidation and methemoglobin formation are both necessary for band 3 clustering: mechanistic insights into human erythrocyte senescence. *Biochemistry* 52 5760–5769. 10.1021/bi400405p 23889086 PMC3914982

[B5] Bardyn M. Rappaz B. Jaferzadeh K. Crettaz D. Tissot J. D. Moon I. (2017). Red blood cells ageing markers: a multi-parametric analysis. *Blood Transfus.* 15 239–248. 10.2450/2017.0318-16 28518051 PMC5448830

[B6] Barras J. P. Moldovanyi A. Wagner S. Koerner K. (1994). [Effect of rejuvenation on the rheologic properties of stored erythrocytes]. *Vasa* 23 305–311. 7817610

[B7] Barshtein G. Goldschmidt N. Pries A. R. Zelig O. Arbell D. Yedgar S. (2017). Deformability of transfused red blood cells is a potent effector of transfusion-induced hemoglobin increment: a study with beta-thalassemia major patients. *Am. J. Hematol.* 92 E559–E560. 10.1002/ajh.24821 28614898

[B8] Barshtein G. Gural A. Manny N. Zelig O. Yedgar S. Arbell D. (2014). Storage-induced damage to red blood cell mechanical properties can be only partially reversed by rejuvenation. *Transfus. Med. Hemother.* 41 197–204. 10.1159/000357986 25053933 PMC4086768

[B9] Basse F. Stout J. G. Sims P. J. Wiedmer T. (1996). Isolation of an erythrocyte membrane protein that mediates Ca^2+^-dependent transbilayer movement of phospholipid. *J. Biol. Chem.* 271 17205–17210. 10.1074/jbc.271.29.17205 8663431

[B10] Bennett-Guerrero E. Veldman T. H. Doctor A. Telen M. J. Ortel T. L. Reid T. S. (2007). Evolution of adverse changes in stored RBCs. *Proc. Natl. Acad. Sci. U.S.A.* 104 17063–17068. 10.1073/pnas.0708160104 17940021 PMC2040393

[B11] Beutler E. Kuhl W. West C. (1982). The osmotic fragility of erythrocytes after prolonged liquid storage and after reinfusion. *Blood* 59 1141–1147. 7082820

[B12] Beutler E. Wood L. (1969). The in vivo regeneration of red cell 2,3 diphosphoglyceric acid (DPG) after transfusion of stored blood. *J. Lab. Clin. Med.* 74 300–304. 5799513

[B13] Bitbol M. Fellmann P. Zachowski A. Devaux P. F. (1987). Ion regulation of phosphatidylserine and phosphatidylethanolamine outside-inside translocation in human erythrocytes. *Biochim. Biophys. Acta* 904 268–282. 10.1016/0005-2736(87)90376-2 3117114

[B14] Bosman G. J. (2013). Survival of red blood cells after transfusion: processes and consequences. *Front. Physiol.* 4:376. 10.3389/fphys.2013.00376 24391593 PMC3866658

[B15] Bosman G. J. Stappers M. Novotny V. M. (2010). Changes in band 3 structure as determinants of erythrocyte integrity during storage and survival after transfusion. *Blood Transfus.* 8 S48–S52. 10.2450/2010.008s 20606749 PMC2897190

[B16] Bosman G. J. Werre J. M. Willekens F. L. Novotny V. M. (2008). Erythrocyte ageing in vivo and in vitro: structural aspects and implications for transfusion. *Transfus. Med.* 18 335–347. 10.1111/j.1365-3148.2008.00892.x 19140816

[B17] Bratosin D. Mazurier J. Tissier J. P. Estaquier J. Huart J. J. Ameisen J. C. (1998). Cellular and molecular mechanisms of senescent erythrocyte phagocytosis by macrophages. A review. *Biochimie* 80 173–195. 10.1016/S0300-9084(98)80024-2 9587675

[B18] Chadebech P. Bodivit G. Razazi K. de Vassoigne C. Pelle L. Burin-des-Roziers N. (2017). Red blood cells for transfusion in patients with sepsis: respective roles of unit age and exposure to recipient plasma. *Transfusion* 57 1898–1904. 10.1111/trf.14170 28568651

[B19] Chadebech P. de Menorval M. A. Bodivit G. Mekontso-Dessap A. Pakdaman S. Jouard A. (2016). Evidence of benefits from using fresh and cryopreserved blood to transfuse patients with acute sickle cell disease. *Transfusion* 56 1730–1738. 10.1111/trf.13636 27184475

[B20] Chung S. M. Bae O. N. Lim K. M. Noh J. Y. Lee M. Y. Jung Y. S. (2007). Lysophosphatidic acid induces thrombogenic activity through phosphatidylserine exposure and procoagulant microvesicle generation in human erythrocytes. *Arterioscler. Thromb. Vasc. Biol.* 27 414–421. 10.1161/01.ATV.0000252898.48084.6a 17110600

[B21] Ciana A. Achilli C. Minetti G. (2017). Spectrin and other membrane-skeletal components in human red blood cells of different age. *Cell. Physiol. Biochem.* 42 1139–1152. 10.1159/000478769 28668958

[B22] Cicha I. Suzuki Y. Tateishi N. Shiba M. Muraoka M. Tadokoro K. (2000). Gamma-ray-irradiated red blood cells stored in mannitol-adenine-phosphate medium: rheological evaluation and susceptibility to oxidative stress. *Vox Sang.* 79 75–82. 10.1046/j.1423-0410.2000.7920075.x 11054044

[B23] D’Alessandro A. D’Amici G. M. Vaglio S. Zolla L. (2012). Time-course investigation of SAGM-stored leukocyte-filtered red bood cell concentrates: from metabolism to proteomics. *Haematologica* 97 107–115. 10.3324/haematol.2011.051789 21993682 PMC3248938

[B24] D’Alessandro A. Gray A. D. Szczepiorkowski Z. M. Hansen K. Herschel L. H. Dumont L. J. (2017). Red blood cell metabolic responses to refrigerated storage, rejuvenation, and frozen storage. *Transfusion* 57 1019–1030. 10.1111/trf.14034 28295356

[B25] D’Alessandro A. Kriebardis A. G. Rinalducci S. Antonelou M. H. Hansen K. C. Papassideri I. S. (2015). An update on red blood cell storage lesions, as gleaned through biochemistry and omics technologies. *Transfusion* 55 205–219. 10.1111/trf.12804 25130459

[B26] D’Alessandro A. Liumbruno G. Grazzini G. Zolla L. (2010). Red blood cell storage: the story so far. *Blood Transfus.* 8 82–88. 10.2450/2009.0122-09 20383300 PMC2851210

[B27] d’Almeida M. S. Jagger J. Duggan M. White M. Ellis C. Chin-Yee I. H. (2000). A comparison of biochemical and functional alterations of rat and human erythrocytes stored in CPDA-1 for 29 days: implications for animal models of transfusion. *Transfus. Med.* 10 291–303. 10.1046/j.1365-3148.2000.00267.x 11123813

[B28] D’Amici G. M. Rinalducci S. Zolla L. (2007). Proteomic analysis of RBC membrane protein degradation during blood storage. *J. Proteome Res.* 6 3242–3255. 10.1021/pr070179d 17585793

[B29] Deplaine G. Safeukui I. Jeddi F. Lacoste F. Brousse V. Perrot S. (2011). The sensing of poorly deformable red blood cells by the human spleen can be mimicked in vitro. *Blood* 117 e88–e95. 10.1182/blood-2010-10-312801 21163923 PMC3062417

[B30] DeVenuto F. Brenneman G. Wilson S. M. (1974). Rejuvenation of human red blood cells during liquid storage. *Transfusion* 14 338–344. 10.1111/j.1537-2995.1974.tb04542.x 4843241

[B31] Dinkla S. Peppelman M. van der Raadt J. Atsma F. Novotny V. M. van Kraaij M. G. (2014). Phosphatidylserine exposure on stored red blood cells as a parameter for donor-dependent variation in product quality. *Blood Transfus.* 12 204–209. 10.2450/2013.0106-13 24120596 PMC4039702

[B32] Dumaswala U. J. Petrosky T. L. Greenwalt T. J. (1992). Studies in red blood cell preservation. 6. Red cell membrane remodeling during rejuvenation. *Vox Sang.* 63 12–15. 10.1111/j.1423-0410.1992.tb01212.x 1413658

[B33] Dumaswala U. J. Wilson M. J. Wu Y. L. Wykle J. Zhuo L. Douglass L. M. (2000). Glutathione loading prevents free radical injury in red blood cells after storage. *Free Radic. Res.* 33 517–529. 10.1080/10715760000301061 11200085

[B34] Dumaswala U. J. Zhuo L. Jacobsen D. W. Jain S. K. Sukalski K. A. (1999). Protein and lipid oxidation of banked human erythrocytes: role of glutathione. *Free Radic. Biol. Med.* 27 1041–1049. 10.1016/S0891-5849(99)00149-5 10569637

[B35] Dzik W. (2008). Fresh blood for everyone? Balancing availability and quality of stored RBCs. *Transfus. Med.* 18 260–265. 10.1111/j.1365-3148.2008.00870.x 18783585

[B36] Enoki Y. Watanabe T. Ohga Y. (1986). Posttransfusional recovery of defective respiratory function of stored blood in dogs. *Jpn. J. Physiol.* 36 1125–1139. 10.2170/jjphysiol.36.1125 3599550

[B37] Erman H. Aksu U. Belce A. Atukeren P. Uzun D. Cebe T. (2016). Gender and chronological age affect erythrocyte membrane oxidative indices in citrate phosphate dextrose adenine-formula 1 (CPDA-1) blood bank storage condition. *Gen. Physiol. Biophys.* 35 343–351. 10.4149/gpb_;2016001 27045670

[B38] Frank S. M. Abazyan B. Ono M. Hogue C. W. Cohen D. B. Berkowitz D. E. (2013). Decreased erythrocyte deformability after transfusion and the effects of erythrocyte storage duration. *Anesth. Analg.* 116 975–981. 10.1213/ANE.0b013e31828843e6 23449853 PMC3744176

[B39] Gao C. Xie R. Yu C. Wang Q. Shi F. Yao C. (2012). Procoagulant activity of erythrocytes and platelets through phosphatidylserine exposure and microparticles release in patients with nephrotic syndrome. *Thromb. Haemost.* 107 681–689. 10.1160/TH11-09-0673 22370875

[B40] Gelderman M. P. Vostal J. G. (2011). Rejuvenation improves roller pump-induced physical stress resistance of fresh and stored red blood cells. *Transfusion* 51 1096–1104. 10.1111/j.1537-2995.2010.02972.x 21133931

[B41] Gevi F. D’Alessandro A. Rinalducci S. Zolla L. (2012). Alterations of red blood cell metabolome during cold liquid storage of erythrocyte concentrates in CPD-SAGM. *J. Proteomics* 76 168–180. 10.1016/j.jprot.2012.03.012 22465715

[B42] Girasole M. Pompeo G. Cricenti A. Longo G. Boumis G. Bellelli A. (2010). The how, when, and why of the aging signals appearing on the human erythrocyte membrane: an atomic force microscopy study of surface roughness. *Nanomedicine* 6 760–768. 10.1016/j.nano.2010.06.004 20603227

[B43] Girasole M. Pompeo G. Cricenti A. Longo G. Boumis G. Bellelli A. (2012). The how, when, and why of the aging signals appearing on the human erythrocyte membrane: an atomic force microscopy study of surface roughness. *Nanomedicine* 6 760–768. 10.1016/j.nano.2010.06.004 20603227

[B44] Godin C. Caprani A. (1997). Effect of blood storage on erythrocyte/wall interactions: implications for surface charge and rigidity. *Eur. Biophys. J.* 26 175–182. 10.1007/s002490050069 9232845

[B45] Heaton A. Keegan T. Holme S. (1989). In vivo regeneration of red cell 2,3-diphosphoglycerate following transfusion of DPG-depleted AS-1, AS-3 and CPDA-1 red cells. *Br. J. Haematol.* 71 131–136. 10.1111/j.1365-2141.1989.tb06286.x 2492818

[B46] Hess J. R. (2010). Red cell storage. *J. Proteomics* 73 368–373. 10.1016/j.jprot.2009.11.005 19914410

[B47] Hod E. A. Brittenham G. M. Billote G. B. Francis R. O. Ginzburg Y. Z. Hendrickson J. E. (2011). Transfusion of human volunteers with older, stored red blood cells produces extravascular hemolysis and circulating non-transferrin-bound iron. *Blood* 118 6675–6682. 10.1182/blood-2011-08-371849 22021369 PMC3242722

[B48] Huang Y. X. Wu Z. J. Mehrishi J. Huang B. T. Chen X. Y. Zheng X. J. (2014). Human red blood cell aging: correlative changes in surface charge and cell properties. *J. Cell. Mol. Med.* 15 2634–2642. 10.1111/j.1582-4934.2011.01310.x 21435169 PMC4373432

[B49] Hunsicker O. Hessler K. Krannich A. Boemke W. Braicu I. Sehouli J. (2018). Duration of storage influences the hemoglobin rising effect of red blood cells in patients undergoing major abdominal surgery. *Transfusion* 10.1111/trf.14627 29665067

[B50] Kekre N. Mallick R. Allan D. Tinmouth A. Tay J. (2013). The impact of prolonged storage of red blood cells on cancer survival. *PLoS One* 8:e68820. 10.1371/journal.pone.0068820 23874777 PMC3712925

[B51] Kiartivich S. Talalak P. Chandanayingyoung D. (1986). The effect of rejuvenation on 2, 3-DPG level of preserved red blood cells. *J. Med. Assoc. Thai.* 69 665–671. 3572281

[B52] Koshkaryev A. Zelig O. Manny N. Yedgar S. Barshtein G. (2009). Rejuvenation treatment of stored red blood cells reverses storage-induced adhesion to vascular endothelial cells. *Transfusion* 49 2136–2143. 10.1111/j.1537-2995.2009.02251.x 19538542

[B53] Kozlova E. Chernysh A. Moroz V. Sergunova V. Gudkova O. Kuzovlev A. (2015). Nanodefects of membranes cause destruction of packed red blood cells during long-term storage. *Exp. Cell Res.* 337 192–201. 10.1016/j.yexcr.2015.07.009 26169694

[B54] Kriebardis A. G. Antonelou M. H. Stamoulis K. E. Economou-Petersen E. Margaritis L. H. Papassideri I. S. (2008). RBC-derived vesicles during storage: ultrastructure, protein composition, oxidation, and signaling components. *Transfusion* 48 1943–1953. 10.1111/j.1537-2995.2008.01794.x 18564399

[B55] Kurach J. D. Almizraq R. Bicalho B. Acker J. P. Holovati J. L. (2014). The effects of rejuvenation during hypothermic storage on red blood cell membrane remodeling. *Transfusion* 54 1595–1603. 10.1111/trf.12490 24224647

[B56] Kuypers F. A. de Jong K. (2004). The role of phosphatidylserine in recognition and removal of erythrocytes. *Cell. Mol. Biol.* 50 147–158. 15095785

[B57] Lang E. Pozdeev V. I. Xu H. C. Shinde P. V. Behnke K. Hamdam J. M. (2016). Storage of erythrocytes induces suicidal erythrocyte death. *Cell. Physiol. Biochem.* 39 668–676. 10.1159/000445657 27442519

[B58] Liu C. Liu X. Janes J. Stapley R. Patel R. P. Gladwin M. T. (2014). Mechanism of faster NO scavenging by older stored red blood cells. *Redox Biol.* 2 211–219. 10.1016/j.redox.2013.12.014 24494195 PMC3909782

[B59] Liumbruno G. Bennardello F. Lattanzio A. Piccoli P. Rossetti G. (2009). Recommendations for the transfusion of red blood cells. *Blood Transfus.* 7 49–64. 10.2450/2008.0020-08 19290081 PMC2652237

[B60] Lockwood W. B. Hudgens R. W. Szymanski I. O. Teno R. A. Gray A. D. (2003). Effects of rejuvenation and frozen storage on 42-day-old AS-3 RBCs. *Transfusion* 43 1527–1532. 10.1046/j.1537-2995.2003.00551.x 14617310

[B61] Luten M. Roerdinkholder-Stoelwinder B. Schaap N. P. de Grip W. J. Bos H. J. Bosman G. J. (2008). Survival of red blood cells after transfusion: a comparison between red cells concentrates of different storage periods. *Transfusion* 48 1478–1485. 10.1111/j.1537-2995.2008.01734.x 18482180

[B62] Lutz H. U. Bogdanova A. (2013). Mechanisms tagging senescent red blood cells for clearance in healthy humans. *Front. Physiol.* 4:387. 10.3389/fphys.2013.00387 24399969 PMC3872327

[B63] Maher A. D. Kuchel P. W. (2003). The Gardos channel: a review of the Ca^2+^-activated K^+^ channel in human erythrocytes. *Int. J. Biochem. Cell Biol.* 35 1182–1197. 10.1016/S1357-2725(02)00310-2 12757756

[B64] Meyer E. K. Dumont D. F. Baker S. Dumont L. J. (2011). Rejuvenation capacity of red blood cells in additive solutions over long-term storage. *Transfusion* 51 1574–1579. 10.1111/j.1537-2995.2010.03021.x 21251004

[B65] Nagababu E. Scott A. V. Johnson D. J. Goyal A. Lipsitz J. A. Barodka V. M. (2016). The impact of surgery and stored red blood cell transfusions on nitric oxide homeostasis. *Anesth. Analg.* 123 274–282. 10.1213/ANE.0000000000001392 27308950

[B66] Nishino T. Yachie-Kinoshita A. Hirayama A. Soga T. Suematsu M. Tomita M. (2009). In silico modeling and metabolome analysis of long-stored erythrocytes to improve blood storage methods. *J. Biotechnol.* 144 212–223. 10.1016/j.jbiotec.2009.08.010 19695295

[B67] Nishino T. Yachie-Kinoshita A. Hirayama A. Soga T. Suematsu M. Tomita M. (2013). Dynamic simulation and metabolome analysis of long-term erythrocyte storage in adenine-guanosine solution. *PLoS One* 8:e71060. 10.1371/journal.pone.0071060 24205395 PMC3796775

[B68] Obrador R. Musulin S. Hansen B. (2015). Red blood cell storage lesion. *J. Vet. Emerg. Crit. Care* 25 187–199. 10.1111/vec.12252 25428860

[B69] Olivieri O. de Franceschi L. de Gironcoli M. Girelli D. Corrocher R. (1993). Potassium loss and cellular dehydration of stored erythrocytes following incubation in autologous plasma: role of the KCl cotransport system. *Vox Sang.* 65 95–102. 10.1111/j.1423-0410.1993.tb02123.x 8212678

[B70] Orbach A. Zelig O. Yedgar S. Barshtein G. (2017). Biophysical and biochemical markers of red blood cells fragility. *Transfus Med Hemother* 44 183–187. 10.1159/000452106 28626369 PMC5473068

[B71] Pantaleo A. Giribaldi G. Mannu F. Arese P. Turrini F. (2008). Naturally occurring anti-band 3 antibodies and red blood cell removal under physiological and pathological conditions. *Autoimmun. Rev.* 7 457–462. 10.1016/j.autrev.2008.03.017 18558362

[B72] Pompeo G. Girasole M. Cricenti A. Boumis G. Bellelli A. Amiconi S. (2012). Erythrocyte death in vitro induced by starvation in the absence of Ca^2+^. *Biochim. Biophys. Acta* 1798 1047–1055. 10.1016/j.bbamem.2010.02.002 20153719

[B73] Prudent M. Tissot J. D. Lion N. (2015). In vitro assays and clinical trials in red blood cell aging: Lost in translation. *Transfus. Apher. Sci.* 52 270–276. 10.1016/j.transci.2015.04.006 25982219

[B74] Raat N. J. Hilarius P. M. Johannes T. de Korte D. Ince C. Verhoeven A. J. (2009). Rejuvenation of stored human red blood cells reverses the renal microvascular oxygenation deficit in an isovolemic transfusion model in rats. *Transfusion* 49 427–434. 10.1111/j.1537-2995.2008.02002.x 19040497

[B75] Racek J. Herynkova R. Holecek V. Jerabek Z. Slama V. (1997). Influence of antioxidants on the quality of stored blood. *Vox Sang.* 72 16–19. 10.1159/000461951 9031495

[B76] Rapido F. Brittenham G. M. Bandyopadhyay S. La Carpia F. L’Acqua C. McMahon D. J. (2017). Prolonged red cell storage before transfusion increases extravascular hemolysis. *J. Clin. Invest.* 127 375–382. 10.1172/JCI90837 27941245 PMC5199711

[B77] Relevy H. Koshkaryev A. Manny N. Yedgar S. Barshtein G. (2008). Blood banking-induced alteration of red blood cell flow properties. *Transfusion* 48 136–146. 10.1111/j.1537-2995.2007.01491.x 17900281

[B78] Safeukui I. Buffet P. A. Deplaine G. Perrot S. Brousse V. Ndour A. (2012). Quantitative assessment of sensing and sequestration of spherocytic erythrocytes by the human spleen. *Blood* 120 424–430. 10.1182/blood-2012-01-404103 22510876 PMC3398764

[B79] Salzer U. Prohaska R. (2001). Stomatin, flotillin-1, and flotillin-2 are major integral proteins of erythrocyte lipid rafts. *Blood* 97 1141–1143. 10.1182/blood.V97.4.1141 11159550

[B80] Santacruz-Gomez K. Silva-Campa E. Alvarez-Garcia S. Mata-Haro V. Soto-Puebla D. Pedroza-Montero M. (2014). An AFM approach of RBC micro and nanoscale topographic features during storage. *World Acad. Sci. Eng. Technol. Int. J.* 8 449–452.

[B81] Shin J. H. Lim K. M. Noh J. Y. Bae O. N. Chung S. M. Lee M. Y. (2007). Lead-induced procoagulant activation of erythrocytes through phosphatidylserine exposure may lead to thrombotic diseases. *Chem. Res. Toxicol.* 20 38–43. 10.1021/tx060114 17226925

[B82] Silva D. C. Jovino C. N. Silva C. A. Fernandes H. P. Filho M. M. Lucena S. C. (2012). Optical tweezers as a new biomedical tool to measure zeta potential of stored red blood cells. *PLoS One* 7:e31778. 10.1371/journal.pone.0031778 22363729 PMC3283675

[B83] Song H. Liu Y. Zhang B. Tian K. Zhu P. Lu H. (2017). Study of in vitro RBCs membrane elasticity with AOD scanning optical tweezers. *Biomed. Opt. Express* 8 384–394. 10.1364/BOE.8.000384 28101425 PMC5231307

[B84] Steiner M. E. Ness P. M. Assmann S. F. Triulzi D. J. Sloan S. R. Delaney M. (2015). Effects of red-cell storage duration on patients undergoing cardiac surgery. *N. Engl. J. Med.* 372 1419–1429. 10.1056/NEJMoa1414219 25853746 PMC5442442

[B85] Stowell C. P. Whitman G. Granger S. Gomez H. Assmann S. F. Massey M. J. (2017). The impact of red blood cell storage duration on tissue oxygenation in cardiac surgery. *J. Thorac. Cardiovasc. Surg.* 153 610–619e2. 10.1016/j.jtcvs.2016.11.029 28027790 PMC5316299

[B86] Svetina S. (2012). Red blood cell shape and deformability in the context of the functional evolution of its membrane structure. *Cell. Mol. Biol. Lett.* 17 171–181. 10.2478/s11658-012-0001-z 22271334 PMC6275855

[B87] Tchir J. D. Acker J. P. Holovati J. L. (2013). Rejuvenation of ATP during storage does not reverse effects of the hypothermic storage lesion. *Transfusion* 53 3184–3191. 10.1111/trf.12194 23581461

[B88] Usry R. T. Moore G. L. Manalo F. W. (1975). Morphology of stored, rejuvenated human erythrocytes. *Vox Sang.* 28 176–183. 10.1111/j.1423-0410.1975.tb02756.x 1119131

[B89] Valeri C. R. Hirsch N. M. (1969). Restoration in vivo of erythrocyte adenosine triphosphate, 2,3-diphosphoglycerate, potassium ion, and sodium ion concentrations following the transfusion of acid-citrate-dextrose-stored human red blood cells. *J. Lab. Clin. Med.* 73 722–733. 5779258

[B90] Valtis D. J. (1954). Defective gas-transport function of stored red blood-cells. *Lancet* 266 119–124. 10.1016/S0140-6736(54)90978-2 13118742

[B91] Verhoeven A. J. Hilarius P. M. Dekkers D. W. Lagerberg J. W. de Korte D. (2006). Prolonged storage of red blood cells affects aminophospholipid translocase activity. *Vox Sang.* 91 244–251. 10.1111/j.1423-0410.2006.00822.x 16958837

[B92] Whillier S. Raftos J. E. Sparrow R. L. Kuchel P. W. (2011). The effects of long-term storage of human red blood cells on the glutathione synthesis rate and steady-state concentration. *Transfusion* 51 1450–1459. 10.1111/j.1537-2995.2010.03026.x 21251007

[B93] Wilkinson D. K. Turner E. J. Parkin E. T. Garner A. E. Harrison P. J. Crawford M. (2008). Membrane raft actin deficiency and altered Ca2+-induced vesiculation in stomatin-deficient overhydrated hereditary stomatocytosis. *Biochim. Biophys. Acta* 1778 125–132. 10.1016/j.bbamem.2007.09.016 17961506

[B94] Wolfe L. C. Byrne A. M. Lux S. E. (1986). Molecular defect in the membrane skeleton of blood bank-stored red cells. Abnormal spectrin-protein 4.1-actin complex formation. *J. Clin. Invest.* 78 1681–1686. 10.1172/JCI112762 3782475 PMC423942

[B95] Woon L. A. Holland J. W. Kable E. P. Roufogalis B. D. (1999). Ca^2+^ sensitivity of phospholipid scrambling in human red cell ghosts. *Cell Calcium* 25 313–320. 10.1054/ceca.1999.0029 10456228

[B96] Yoshida T. AuBuchon J. P. Dumont L. J. Gorham J. D. Gifford S. C. Foster K. Y. (2008). The effects of additive solution pH and metabolic rejuvenation on anaerobic storage of red cells. *Transfusion* 48 2096–2105. 10.1111/j.1537-2995.2008.01812.x 18631166

